# Families' costs form a considerable part of total costs in bronchiolitis care

**DOI:** 10.1002/hsr2.593

**Published:** 2022-04-26

**Authors:** Emilia Mäntynen, Sauli Palmu, Paula Heikkilä

**Affiliations:** ^1^ Faculty of Medicine and Health Technology Tampere University Tampere Finland; ^2^ Tampere Centre for Child, Adolescent and Maternal Health Research, Faculty of Medicine and Health Technology Tampere University and Tampere University Hospital Tampere Finland

**Keywords:** bronchiolitis, cost, cost analysis, infant, hospitalization

## Abstract

**Background and aim:**

The burden of bronchiolitis is remarkable due to high morbidity in infants. The aim of this study was to evaluate bronchiolitis‐associated costs for patients' families and the healthcare system.

**Methods:**

This retrospective, descriptive study included 136 infants under 12 months of age treated at Tampere University Hospital, Finland, between October 1, 2018 and March 31, 2020, with bronchiolitis as the main diagnosis. The data consists of patient background and medical information and of estimated costs for the families and for the healthcare system. The data were collected from the hospital's electronic patient files and registries and were analyzed with descriptive statistical analyzes using SPSS v. 26 software.

**Results:**

The total median costs associated with bronchiolitis from the perspective of families and healthcare were €16,205 per patient if intensive care was needed and €2266 per patient treated only on the ward. The median costs for the families were €461 and €244, respectively, and for the healthcare system, they were €15,644 and €2019.

**Conclusion:**

The majority of the total costs for treatment were due to healthcare costs and only 10% of costs were targeted at families. Bronchiolitis‐associated total median costs were 7.2 times higher and the families' costs were 1.9 times higher if intensive care was needed instead of treatment on the ward only.

## 1 INTRODUCTION

Infectious diseases, such as respiratory tract infection and diarrhea, are the leading reasons for morbidity during early childhood.^1^, ^2^ This study is focused on bronchiolitis, which is a seasonal lower respiratory tract infection that causes significant use of healthcare services. Bronchiolitis is the most common cause of hospitalization among children under 12 months of age and around 1%–3% of all infants with bronchiolitis are hospitalized each year.^3^, ^4^ Approximately 2%–6% of those hospitalized infants are treated in the pediatric intensive care unit (PICU).^5^, ^6^ Premature infants have considerably higher admission rates and longer duration of episodes than term infants.^7^

Although hospitalizations for bronchiolitis have decreased in the United States, healthcare costs have increased 63% between 2000 and 2016, leading to annual total costs of US$734 million.^8^ Earlier studies have shown that healthcare system costs have varied between €1800 and €4000 per one bronchiolitis hospitalization,^9^, ^10^, ^11^, ^12^ increasing to roughly €8000–€16,800 when intensive care is needed.^9^, ^13^ Moreover, the costs have been shown to be higher for premature infants and prematurity leads to significantly higher healthcare costs during the following 12 months.^14^

Although the burden of bronchiolitis is considerable for both families and society, there are only a few studies from their perspectives. According to one German study, total socioeconomic costs were €3700 per hospitalization and the parental cost was €87.^12^ According to a Dutch study, 15% should be added to the hospital‐related costs to cover costs from the perspective of families and society.^15^ As these two studies were published nearly two decades ago, up‐to‐date information on bronchiolitis‐associated costs from a wider perspective is needed.

The aim of this retrospective study was to evaluate costs associated with bronchiolitis hospitalization from the perspectives of families and healthcare organizations, that is, the hospital, as well as to separately evaluate the costs for treating infants in PICUs or on wards only.

## 2 METHODS

This was a retrospective chart review describing the cost of bronchiolitis hospitalization in infants treated at Tampere University Hospital (Tays), Finland. Tays provides secondary and tertiary healthcare for ~4400 infants under 1 year of age living in Tampere and its surroundings, both in urban and rural areas. The healthcare system in Finland is universal and tax‐funded. The copayment for the patients or the families is low, meaning that healthcare services are affordable for all patients. Public or private primary healthcare provides only preventive or outpatient healthcare for children, which means that all patients in this area who need inpatient care are treated in Tays.

All infants under 12 months requiring inpatient treatment between October 1, 2018 and March 31, 2020, for bronchiolitis (International Classification of Disease‐10 diagnosis codes J21.0, J21.8, and J21.99) were included in the study. Bronchiolitis was defined as the first expiratory difficulty during an acute viral lower respiratory tract infection in infants under 12 months of age. The data were collected from electronic patient files and registries at Tays.

One of the authors (EM) collected the background and medical data in a structured case report form, including the details of gender, age on admission, number of siblings, length of stay (LOS) in days on the ward and in the PICU, number of family visits during hospitalization, the distance of travel between hospital and patients' home, private doctor visits, ambulance transfers, primary and secondary emergency department (ED) visits and readmissions within 4 weeks of the index hospitalization.

The cost data consists of the costs from the perspectives of families and healthcare, that is, the hospital. The estimate of families' costs included hospital fees (paid by the families in addition to the reimbursement paid by municipalities), travel costs, additional meal costs due to hospitalization, private doctor fees, and ambulance fees; children's visits to primary healthcare facilities are free of charge in Finland and thus were not included. The detailed cost estimations for the families are presented in Table 1 and a more detailed description of the cost estimation methods is presented in the Supporting Information.

**Table 1:** Details of the costs used in the estimation of families' costs for bronchiolitis requiring inpatient treatment in infants

	Cost in €
Hospital fee (per inpatient day)	47.9
Hospital fee (per outpatient visit)	41.2
Ambulance transfer^a^	25
Visit of a private pediatrician	96
Travel costs, €/km	0.43
Additional meal cost (per hospitalization day)	10.75

^a^ Cost for patient. Cost for municipality is considerably higher.

The healthcare costs were defined as the costs and reimbursement caused by the hospitalization of bronchiolitis and were collected from Tampere University Hospital's cost‐per‐patient software. The detailed costs consist of daily costs of treatment, laboratory charges, imaging charges and other costs due to treatment. In Finland, hospital reimbursement is paid by the municipalities.

The costs are presented in Euros (€). Costs were not adjusted for inflation due to the short study period (19 months) and low inflation during that time. When comparing results to earlier studies, the annual mean exchange rate for the publication year was used: €1 = $0.8956 (in 2001), €1 = $0.9456 (in 2002), and €1 = $1.3281 (in 2013).^16^

This study was performed with the permission of the Research Director of the hospital. As the study is based on hospital registers and patients were not contacted, no approval from the ethics committee was required.

### 2.1 Statistics

SPSS statistics version 26 (IBM Corp) was used to perform the descriptive analysis. The data were presented as numbers and percentiles. In addition, nonnormally distributed continuous data were presented with median and either interquartile ranges (IQR) or minimum and maximum values. Spearman's correlation was used to analyse the correlation between hospitalization costs and the LOS.

## 3 RESULTS

**Figure 1:**
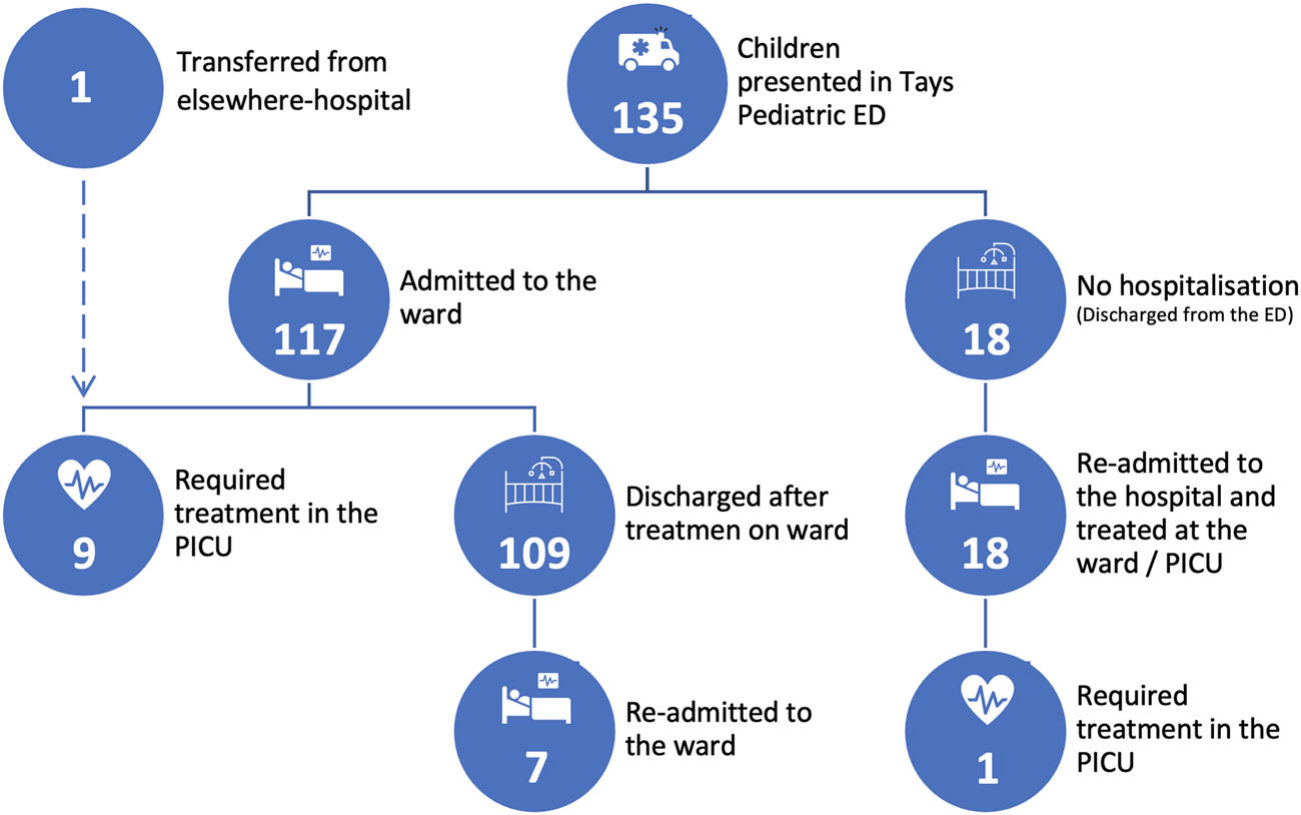
Description of patient flow of 136 infants treated for bronchiolitis as inpatients in Tampere University Hospital between October 1, 2018 and March 31, 2020

A total of 136 infants under 12 months of age treated between October 1, 2018 and March 31, 2020 for bronchiolitis were included in the study. All patients presented at the ED, except one who was admitted directly to intensive care from a secondary care hospital (Figure 1). The total number of infants visiting the ED was 135, with 117 being admitted on their primary visit and 7 readmitted later. Eighteen infants who were not admitted on their primary visit returned for one or more secondary visits and eventually all required inpatient treatment. One of them needed intensive care (Figure 1).

All infants included in this study were young; the median age was 3.0 months (IQR 1.0–5.0). Most of the patients were boys (53.7%) and the most common number of siblings was one (range 0–6; Table 2). The median LOS on the ward for a primary episode was 2.0 days (0.5–6.5), for a secondary episode it was 3.0 days (range 0.5–8.5), and the median LOS in the PICU was 2.5 days (range 1.0–29.0; Table 2).

**Table 2:** Characteristics of the 136 infants treated for bronchiolitis in Tampere University Hospital between October 1, 2018 and March 31, 2020, number of outpatient visits, and LOS in hospital in days

	Median
	(min–max)
	IQR25–IQR75
	*n* = 136
Age on admission (months)	3.0
	(0–11)
	1.0–5.0
Sex (male) *n (%)*	73 (53.7)
Number of siblings, *n* = 113	1
	(0–6)
	1–2
PICU‐treated patients, *n (%)*	10 (7.4)
ED visits	
First visit, *n* = 135	1
	(1–1)
Secondary visit *n* = 26	1
	(1–3)
Ward LOS	
Primary episode, *n* = 118	2.0
	(0.5–8.5)
	1.0–4.0
Secondary episode, *n* = 18	3.0
	(0.5–6.5)
	1.5–4.0
PICU LOS	
Primary episode, *n* = 9	2.5
	(1.0–29.0)
	2.0–5.0
Secondary episode, *n* = 1	2.5
Total LOS	
Primary episode, *n* = 118	2.0
	(0.5–32.5)
	1.0–4.0
Secondary episode, *n* = 25	3.0
	(0.5–6.5)
	1.5–4.3

Abbreviations: ED, emergency department; IQR, interquartile range; LOS, length of stay; PICU, pediatric intensive care unit.

The total median costs associated with bronchiolitis hospitalization were €16,205 for those treated in the PICU and €2266 for those treated only in the ward (Table 3). The median costs for the families were €461 for patients treated in the PICU and €244 for patients treated in the ward (Table 3). The median healthcare cost across the study population was €2422. Patients treated in the PICU had higher median healthcare costs than those patients treated on the ward only: €15,644 versus €2019, respectively. The higher hospitalization costs were strongly correlated with longer LOS (*r* = 0.912, *p* < 0.001). The median reimbursement was lower than the median costs both for those treated in the PICU and those treated only in the ward. Most of the hospitalization costs consisted of the daily cost of treatment, with other relevant cost sources being imaging and laboratory costs. Median imaging costs were €56 (min €43, max €1988), with imaging being conducted for 31 infants. Laboratory costs were higher among infants treated in the PICU than among those treated on the ward: €1155 versus €70, respectively (Table 3).

**Table 3:** Costs (in euros) for families, hospital reimbursement details, and cost of treatment for the hospitalization of 136 infants with bronchiolitis treated in Tampere University Hospital as inpatients between October 1, 2018 and March 31, 2020

	Patients treated on the ward only (*n* = 126)	Patients treated in the PICU and at the ward (*n* = 10)	All patients (*n* = 136)
	Median	Median	Median
	(IQR25; IQR75)	(IQR25; IQR75)	(IQR25; IQR75)
	min–max	min–max	min–max
Total costs (for families and healthcare)	2266	16,205	2637
	(1610; 3485)	(12,106; 33,356)	(1,683; 3,704)
	878–14,765	7672–444,624	878–444,624
Total family costs	244	461	265
	(196; 345)	(397; 583)	(199; 360)
	98–1002	352–707	98–1002
Hospital fees in the ED	41	41.2	41
	(41; 41)	(41; 41)	(41; 41)
	41–165	0–82	0–165
Hospital fees on the ward or in the PICU	96	287	96
	(48; 192)	(228; 335)	(48; 192)
	48–335	144–335	48–335
Total travel costs	41	68	43
	(24; 83)	(34; 233)	(24; 93)
	6–626	24–241	6–626
Meal costs (*n* = 40)	32	54	32
	(16; 43)	(32; 97)	(22; 43)
	11–86	32–97	11–97
Hospital reimbursement	1969	14,340	1986
	(1643; 3109)	(13,207; 28,925)	(1643; 3317)
	869–14,201	8008‐444,308	869–444,308
Hospitalization costs	2019	15,644	2422
	(1415; 3148)	(11,697; 32,820)	(1420; 3301)
	761–14,183	7169–444,222	761–444,222
Hospitalization costs per treatment day	1007	2665	1095
	(766; 1688)	(1965; 4124)	(777; 1796)
	411–5944	1602–13,671	411–13,671
Laboratory costs (*n* = 77)	70	1155	80
	(26; 95)	(715; 2239)	(28; 148)
	9–699	402‐11,187	9–11,187
Imaging costs (*n* = 31)^a^	44	196	56
	(43; 56)	(112; 560)	(44; 112)
	43–112	112–1988	43–1988

Abbreviations: ED, emergency department; IQR, interquartile range; PICU, pediatric intensive care unit.

^a^ (8/10 PICU, 23/126 ward only).

## 4 DISCUSSION

In this retrospective descriptive study, we evaluated the costs associated with bronchiolitis hospitalizations from the perspectives of families and healthcare, that is, the hospital. Our main findings were that the median total cost per patient was €2637 but the range was remarkably wide, €878–444,624, and that the families' costs were ~10% of total median costs. Patients treated in the PICU had considerably higher costs than those treated only on the ward.

Only a few studies have evaluated bronchiolitis‐associated costs for families. In a French study including 777 hospitalized infants with bronchiolitis, the average total cost for the society was €1919 per patient. The majority of the costs were due to hospitalization costs (€1418; 74%) and the rest were due to families' costs (€421; 22%) and due to employers (€80; 4%).^17^ One German study including 77 hospitalized infants with bronchiolitis reported €81 costs for parents in 2005.^12^ The study defined parental costs as a direct nonmedical cost that included supplemental childcare, transportation, absence from work, and additional expenses. Parental costs were collected in this German study by telephone interviews within 4 weeks of hospitalization. Total costs, which included direct and indirect medical costs, as well as direct and indirect nonmedical costs, were €3551 per hospitalized bronchiolitis case.^12^ An older questionnaire study from the Netherlands, with 73 infants hospitalized with bronchiolitis, reported family costs of €329 ($295, 2001) when defined as workdays lost, costs for traveling and family doctor's consultation.^15^ Total socioeconomic costs for bronchiolitis hospitalization were €2,456 ($2200) in 2001. The study in question argued that 15% should be added to the hospital‐related costs to cover family and socioeconomic costs.^15^ A more recent publication from Spain assessed families' direct and indirect costs prospectively but collected data only for ED treatment.^18^ Diagnostic procedures, time spent in the ED and medication were included as direct costs, and loss of parents' work hours, supplementary babysitting, travels, and meals were included as indirect costs. These mean costs were €213 and €36, respectively.^18^

Our results support previous evidence that family costs associated with bronchiolitis hospitalization are a considerable expense for families. The median family cost was €244 per hospitalized infant with bronchiolitis treated on the ward and €461 per hospitalized infant with bronchiolitis treated in the PICU. However, the family costs ranged up to €1000 depending on the LOS in hospital and the distance of travel. Total travel expenses formed a substantial part of total family costs, alongside hospital fees. Direct comparison of family costs with those of previous studies was not possible because of the different parameters used.

In a French retrospective cohort analysis including 525 infants requiring respiratory support, researchers found that children treated in the PICU between 2006 and 2010 because of bronchiolitis with nasal continuous positive airway pressure had mean hospital costs of €16,800.^13^ In another retrospective French study using data for infants hospitalized during their first year of life because of respiratory syncytial virus infection, the mean hospitalization cost per patient was €3973.^10^ A retrospective study conducted in our hospital in Finland including 240 infants with bronchiolitis reported a mean hospitalization reimbursement of €8061 when patients were treated in the PICU and €1834 when patients were treated on the ward in 2015.^9^ Moreover, a retrospective study from the United States including 5334 infants hospitalized with bronchiolitis reported a mean hospitalization cost of €4163 ($3937) in 2002.^11^

Previous findings are in line with our results that the median hospitalization costs were €2019, €15,644 and €2422 for patients treated only on the ward, for patients treated in the PICU and for all patients, respectively. The hospitalization costs seem to have increased in our hospital since 2015, which may be partly due to inflation or changes in both internal and external prices, but also perhaps due to a different approach used in the evaluation of costs in the studies. As shown in the present study, the reimbursement is lower than hospitalization costs, which is mostly the result of political decisions. Differences between earlier studies may be explained by differences among healthcare organizations and healthcare financing in countries as well as by different ways of estimating the costs in the studies. However, the results are remarkably different when comparing the costs between the high‐income and low‐income countries,^19^ due to different general price levels. The comparison between low‐ and high‐income countries was beyond the scope in this study, but would be an interesting aim for a new study, to evaluate the differences between bronchiolitis costs all over the world.

Previous studies have found that by following the local or well‐established bronchiolitis guidelines, both the costs associated with bronchiolitis hospitalization and the use of unnecessary—and often harmful—treatments have decreased. A Finnish study published in 2018 demonstrated a substantial decrease in costs related to bronchiolitis medications after the publication of national Current Care Guidelines advising against racemic adrenaline use in treatment.^20^ In addition, a US retrospective study including 267 patients with bronchiolitis between 2009 and 2012 reported that a higher adherence level in bronchiolitis clinical pathway recommendations was associated with lower treatment costs for bronchiolitis patients.^21^ According to a Colombian study assessing cost‐effectiveness in the utilization of “good practice” published in 2019, both readmissions within 10 days and costs were lower when following a bronchiolitis clinical practice guideline.^22^ Moreover, a US intervention study including 2929 infants found that after bronchiolitis guideline implementation, the total mean cost per patient was reduced by €148 ($197, 2013); in addition, a reduction of 23% in chest radiographs was observed.^23^ In another intervention study of bronchiolitis, guideline implementation by the EDs and urgent care centers proved that albuterol use, chest x‐rays, virus testing and costs were reduced after guideline implementation.^24^ It is evident from these studies that although the cost related to bronchiolitis treatment is substantial, implementing treatment guidelines may reduce costs in addition to improving the treatment.

## 5 LIMITATIONS

The main limitation of our study was its retrospective nature. Families' costs were probably underestimated due to the study design; not all the information needed was available from electronic patient files. As an example, the information regarding parents' working status was unavailable, and consequently, loss of income could not be estimated. Moreover, we could not estimate the need for supplemental childcare, which could sum up to a significant cost for parents. In addition, we did not have information on parents' money consumption and, accordingly, the meal costs were most likely underestimated. For lower‐income families, in particular, having a child hospitalized can be a substantial extra expense. On the other hand, we tried to estimate as well as possible every cost associated with bronchiolitis treatment, for example, travel allowances and meal allowances. We did not use any validated questionnaires to extract data from the hospital registers, but all data that was available was collected. Thus, this study adds necessary information about costs from the family's perspective.

Another limitation is that the number of patients treated in the PICU was relatively small. Moreover, one of the patients had significantly higher costs than the others due to a rare underlying condition and very expensive treatment needed during an extended hospitalization. To minimize the effect, we used the median value instead of the mean value. In addition, we also verified this in an *ad hoc* analysis by removing this patient, and the results did not change.

## 6 CONCLUSION

The majority of the bronchiolitis‐associated costs were due to healthcare costs both in those treated in the intensive care unit (97%) and for those treated only on the ward (89%). Bronchiolitis‐associated total median costs were 7.2 times higher if intensive care was needed instead of treatment on the ward only. Approximately 10% of the total costs of all patients were targeted at the families, and the families' costs were 1.9 times higher if intensive care was needed.

## AUTHOR CONTRIBUTIONS

*Design and conceptualization*: Paula Heikkilä and Sauli Palmu. *Formal analysis*: Emilia Mäntynen and Paula Heikkilä. *Interpretation of the data*: Emilia Mäntynen, Sauli Palmu and Paula Heikkilä. *Funding acquisition*: Paula Heikkilä. *Writing–review and editing*: Sauli Palmu and Paula Heikkilä. *Writing–original draft*: Emilia Mäntynen. All authors have read and approved the final version of the manuscript.

## CONFLICTS OF INTEREST

The authors declare no conflicts of interest.

## ETHICAL STATEMENT

This study was approved by the Research Director of the Tampere University Hospital (R19631S). According to Finnish law, neither approval from the Ethics Committee nor informed consent from the subjects and/or their guardians were not required for this retrospective register‐based study. This study was carried out in accordance with the Declaration of Helsinki. All patient data were managed and conformed to the European Union's General Data Protection Regulation (GDPR) and the data security legislation of Finland.

## TRANSPARENCY STATEMENT

Paula Heikkilä affirms that this manuscript is an honest, accurate, and transparent account of the study being reported; that no important aspects of the study have been omitted; and that any discrepancies from the study as planned have been explained.

## Supporting information

Supporting information

## Data Availability

The anonymous data that support the findings of this study are available on request from the corresponding author. The data are not publicly available due to privacy or ethical restrictions.
