# Supplementary material for: Families' costs form a considerable part of total costs in bronchiolitis care
Source: Health Sci Rep. 2022 Apr 26;5(3):e593. doi: 10.1002/hsr2.593 (PMC9059178; doi:10.1002/hsr2.593)
Supplement: Supplementary file 1 — Supporting information [file HSR2-5-e593-s001.docx]

**Supplementary Material**

**The cost estimation details from the families’ perspective**

The ED cost is €41.20 per visit,^1^ and the hospital fee is €47.9 for each day on the ward or in the PICU (Table 1), that fee being charged from the start of every 24 hours. Hospital fees were further multiplied by LOS. In Finland, the family must pay for the first seven inpatient days only if the child is under 18 years old, with any subsequent days being paid by the municipalities.

Meal costs were estimated at €10.75 per day^2^ (Table 1), which is equal to the meal-imbursement recommended by the Finnish tax authorities. For women who were breastfeeding their hospitalised infant, the meal cost was not calculated because they were eligible for a meal free of charge from the hospital. Furthermore, the daily cost was multiplied by LOS or number of family visits to estimate the additional meal costs for families. If the LOS was shorter than the number of family visits, the LOS in days was used.

Travel costs were estimated at €0.43 per kilometre (Table 1), as recommended by the tax authorities, multiplied by the distance from home in kilometres and the number of visits.^15^ The ambulance fee for the families was €25^3^ if transportation by ambulance was needed, with the actual cost being covered by society. The private doctor fee was estimated at €96 per visit, the average private doctor cost (max 20 min) reported by the Social Insurance Institution’s report.^4^

**The cost estimation details from the hospital’s perspective**

The data from the cost-per-patient programme consisted of hospital reimbursements paid by municipalities in euros. This consisted of the detailed costs of hospital care (including estimation of daily patient care, housekeeping and building maintenance, employee costs, feeding and medicinal costs), imaging and laboratory services in hospital, specialist consultations, possible intensive care or operation theatre costs, or costs associated with other specialities in hospital such as dieticians or physiotherapy per each hospitalisation episode.

**References**

1. TAYS 2020: Asiakasmaksut. Instruction. Updated 25.9.2020. Customer fees in Tampere University Hospital (in Finnish only). <https://www.tays.fi/asiakasmaksut>

2. Tax administration: Kilometre and per diem allowances. Instruction. Updated 26.11.2018. <https://www.vero.fi/en/individuals/vehicles/kilometre_and_per_diem_allowances/>

3. The Social Insurance Institution: Reimbursement amount and copayment. Instruction. Updated 5.12.2018. <https://www.kela.fi/web/en/reimbursements-of-travel-costs-copayment>

4. The Social Insurance Institution: Average fees charged for private-sector medical services. Report. Updated 2019. <http://raportit.kela.fi/ibi_apps/WFServlet?IBIF_ex=NIT236AL&YKIELI=E>
